# Possible Relevance of PNPLA3 and TLL1 Gene Polymorphisms to the Efficacy of PEG-IFN Therapy for HBV-Infected Patients

**DOI:** 10.3390/ijms21093089

**Published:** 2020-04-27

**Authors:** Hirayuki Enomoto, Nobuhiro Aizawa, Kunihiro Hasegawa, Naoto Ikeda, Yoshiyuki Sakai, Kazunori Yoh, Ryo Takata, Yukihisa Yuri, Kyohei Kishino, Yoshihiro Shimono, Noriko Ishii, Tomoyuki Takashima, Takashi Nishimura, Hiroki Nishikawa, Yoshinori Iwata, Hiroko Iijima, Shuhei Nishiguchi

**Affiliations:** 1 Division of Hepatobiliary and Pancreatic Disease, Department of Internal Medicine, Hyogo College of Medicine Division of Hepatobiliary and Pancreatic Disease, Department of Internal Medicine, Hyogo College of Medicine, Nishinomiya, Hyogo 663-8501, Japan; nobu23hiro@yahoo.co.jp (N.A.); hiro.red1230@gmail.com (K.H.); nikeneko@hyo-med.ac.jp (N.I.); sakai429@hyo-med.ac.jp (Y.S.); mm2wintwin@ybb.ne.jp (K.Y.); chano_chano_rt@yahoo.co.jp (R.T.); gyma27ijo04td@gmail.com (Y.Y.); hcm.kyohei@gmail.com (K.K.); yoshihiro19870729@yahoo.co.jp (Y.S.); ishinori1985@yahoo.co.jp (N.I.); tomo0204@yahoo.co.jp (T.T.); tk-nishimura@hyo-med.ac.jp (T.N.); nishikawa_6392_0207@yahoo.co.jp (H.N.); yo-iwata@hyo-med.ac.jp (Y.I.); hiroko-i@hyo-med.ac.jp (H.I.); nishiguc@hyo-med.ac.jp (S.N.); 2 Ultrasound Imaging Center, Hyogo College of Medicine, Nishinomiya, Hyogo 663-8501, Japan; 3 Center for Clinical Research and Education, Hyogo College of Medicine, Nishinomiya, Hyogo 663-8501, Japan

**Keywords:** hepatic steatosis, single nucleotide polymorphism, HBs antigen, sequential therapy

## Abstract

Lifestyle changes have led to an increase in the number of patients with nonalcoholic fatty liver disease (NAFLD). However, the effects of NAFLD-associated single-nucleotide gene polymorphisms (SNPs) in HBV-infected patients have not been adequately investigated. Methods: We investigated the association of the NAFLD-related SNPs patatin-like phospholipase domain-containing protein 3 (PNPLA3; rs738409), transmembrane 6 superfamily member 2 (TM6SF2; rs58542926), 17-beta hydroxysteroid dehydrogenase 13 (HSD17B13; rs72613567, rs6834314 and rs62305723), membrane-bound O-acyltransferase domain containing 7 (MBOAT7; rs641738) and glucokinase regulatory protein (GCKR; rs1260326) with the presence of histologically proven hepatic steatosis (HS) in HBV-infected patients (*n* = 224). We also investigated tolloid-like 1 (TLL1) SNP (rs17047200), which has been reported to be involved in the disease progression in Japanese NAFLD patients, and evaluated the association of HS and various SNPs with the treatment efficacy of pegylated-interferon (PEG-IFN) monotherapy following nucleotide/nucleoside (NA) treatment (NA/PEG-IFN sequential therapy; *n* = 64). Among NAFLD-associated SNPs evaluated, only the PNPLA3 SNP was significantly associated with the presence of hepatic steatosis in a total of 224 HBV-infected patients (P = 1.0 × 10^−4^). Regarding the sequential therapy, PNPLA3 SNP and TLL1 SNP were related to the treatment efficacy, and patients without minor alleles of these SNPs showed favorable results with a high virologic response and significant reduction in their HBsAg titer. A multivariate analysis showed that HBeAg positivity (odds ratio 5.810, *p* = 0.016) and the absence of a risk allele in PNPLA3 and TLL1 SNPs (odds ratio 8.664, *p* = 0.0042) were significantly associated with treatment efficacy. The PNPLA3 SNP might be associated with the presence of HS, and the combination of the PNPLA3 and TLL1 SNPs might be related to the efficacy of PEG-IFN monotherapy following NA treatment.

## 1. Introduction

Hepatitis B virus (HBV) infection is a major cause of chronic liver disease (CLD) [1,2,3]. The management of HBV-infection is extremely important, particularly in areas with a high prevalence of HBV, such as Asia [3]. Many viral and host factors can affect the clinical manifestations of HBV-infection, and HBV-infected patients show varied clinical courses.

Lifestyle changes have led to an increase in the number of patients with metabolic disorder-related diseases, including nonalcoholic fatty liver disease (NAFLD)/hepatic steatosis (HS). Although NAFLD/HS itself is a major cause of CLD [4,5], it can also develop in patients who already have various CLDs. HS is reported to be a major factor causing ALT elevation in HBV-infected patients with a low viral load [6,7], and a meta-analysis suggested that the presence of HS may influence the efficacy of antiviral therapy, especially in nucleotide/nucleoside analogue (NA) treatment; this effect is less commonly observed in patients undergoing pegylated-interferon (PEG-IFN) treatment [8].

Although HS is closely related to patients’ lifestyle, some genetic characteristics are known to be associated with the development of NAFLD/HS [9]. Among NAFLD-associated genetic polymorphisms, patatin-like phospholipase domain-containing protein 3 (PNPLA3) is regarded as the most important, as the single-nucleotide polymorphism (SNP) has been shown to be universally associated with the development of NAFLD [9]. Recent studies have suggested that, in addition to influencing several clinical features in patients with NAFLD [9], the PNPLA3 SNP is also related to the development of HS in HBV-infected patients [10,11,12]. HCV infection (typically genotype 3 virus) is known to cause HS, and the role of HS in patients with viral hepatitis has been mainly studied in patients with HCV infection [13,14,15,16]. In contrast to HCV infection, HBV infection is less relevant to the development of HS [17], so HBV-infected patients may be a good model for assessing the clinical role of NAFLD-associated SNPs in CLDs. However, the impact of NAFLD-related SNPs on antiviral treatment in HBV-infected patients has been poorly investigated. 

In the present study, we evaluated the association of NAFLD-associated SNPs with the presence of histologically-proven HS in HBV-infected patients and their influence on the efficacy of antiviral treatment, including a reduction in the quantitative HBV surface antigen (HBsAg) titer.

## 2. Results

### 2.1. The Association of NAFLD-Associated Gene Polymorphisms with HS in HBV-Infected Patients

As mentioned in the “Patients and Methods” section, we consecutively enrolled a total of 224 patients who received a liver biopsy and evaluated seven genetic polymorphisms associated with the development of NAFLD: PNPLA3 rs738409, transmembrane 6 superfamily member 2 (TM6SF2) rs58542926 [18], 17-beta hydroxysteroid dehydrogenase 13 (HSD17B13) rs72613567, HSD17B13 rs6834314, HSD17B13 rs62305723, membrane-bound O-acyltransferase domain containing 7 (MBOAT7) rs641738 and glucokinase regulatory protein (GCKR) rs1260326 [19,20,21]. The basic characteristics of the patients are shown in Table 1.

With regard to the PNPLA3 SNP, the frequency of HS was significantly different among the groups with different genotypes. In addition, the G (minor) allele frequency in patients with HS was significantly higher than that in patients without HS (Table 2). Furthermore, the genetic variants showed a statistically significant association with the presence of HS (Table 3). However, the SNPs of the remaining genes (TM6SF2, HSD17B13, MBOAT7 and GCKR) were not related to the frequency of HS in HBV-infected patients of our cohort (Table 2). The HSD17B13 rs72613567 SNP was reported to be highly linked with rs6834314 SNP (r^2^ = 0.94) [20], and the genotypes of two HSD17B13 SNPs (rs72613567 and rs6834314) were completely matched in all HBV-infected patients of the current study.

### 2.2. The Association of HS and NAFLD-Associated SNPs with the Response to PEG-IFN Therapy

We next assessed the impact of histologically confirmed HS and NAFLD-associated SNPs in patients receiving PEG-IFN therapy. With regard to the virologic response (VR) after 48 weeks of PEG-IFN therapy, the overall response rates at 24 and 48 weeks after the off-treatment phase were 29/64 (45.3%) and 21/64 (32.8%), respectively. Patients with HS showed a lower VR rate at 48 weeks after the off-treatment phase than those without HS, although the difference did not reach a statistical significance (Table 4). Regarding the PNPLA3 SNP, patients with the CC type had a higher response rate than those with other types, and the difference reached statistical significance (Table 4 and Supplementary Figure S1). The PNPLA3 SNP also showed a statistically significant association with the VR ratios (Supplementary Table S1). In addition, the responders and non-responders showed significant differences in two factors: the effects of preceding NA treatment and HBeAg positivity. However, unlike HCV treatment [22], the interleukin 28B (IL28B) SNP (rs8099917) was not shown to be related to the efficacy of anti-HBV therapy in our cohort. Several NAFLD-related gene SNPs other than PNPLA3 SNP were also not significantly associated with the treatment response to PEG-IFN therapy (Table 4).

**Figure 1:**
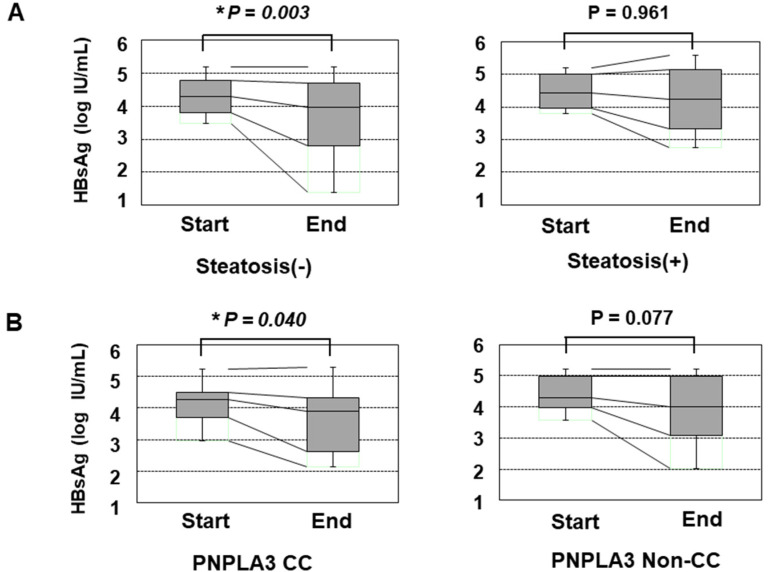
The transition of the HBsAg values in patients over 48 weeks of PEG-IFN. (**A**) a comparison of the HBsAg values at the initiation and termination of PEG-IFN treatment. The left and right panels show the HBsAg values of patients without HS (*n* = 48) and with HS (*n* = 16), respectively. (**B**) the association of the PNPLA3 SNP with the decrease in the HBsAg titer in response to PEG-IFN treatment. The left and right panels show the HBsAg values of patients with the CC type (*n* = 17) and non-CC types (*n* = 47), respectively. *: *p* < 0.05.

We also assessed the reduction in the HBsAg titer in response to PEG-IFN treatment. Before the initiation of PEG-IFN therapy, the presence of HS and the types of PNPLA3 SNPs did not significantly affect the HBsAg titer (Supplementary Figure S2). However, patients without HS showed a significant reduction in the HBsAg titer at the end of PEG-IFN treatment, while those with HS did not show any such reduction (Figure 1A). Furthermore, the patients with the CC type showed a statistically significant reduction in HBsAg, while those with the non-CC types did not (Figure 1B). Although the HBsAg titers of the patients with non-CC types did not show a significant reduction, some patients with the CG type responded to PEG-IFN treatment and showed reduced HBsAg values, while the reduction in HBsAg values was limited in patients with the GG type (Supplementary Figure S3).

### 2.3. Role of the TLL1 SNP and Its Combination with the PNPLA3 SNP

**Figure 2:**
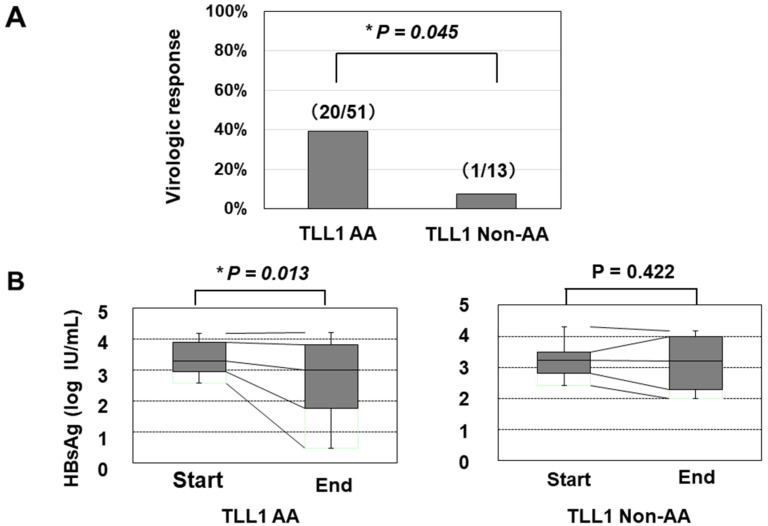
The association of the TLL1 SNP with the virologic response and decrease in the HBsAg titer in response to PEG-IFN treatment. (**A**) The response rates in patients with the AA and non-AA types of the TLL1 SNPs were 53.8% (20/51) and 9.1% (1/13), respectively (*: *p* < 0.05). (**B**) the association of the TLL1 SNP with the decrease in the HBsAg titer in response to PEG-IFN treatment. The HBsAg values at the initiation and termination of PEG-IFN treatment were compared. *: *p* < 0.05.

We further determined the TLL1 gene SNP rs17047200, which was shown to be related to the development of hepatocellular carcinoma (HCC) in HCV-eradicated patients [23,24], since the combination of PNPLA3 and TLL1 SNPs was recently suggested to be associated with disease progression in Japanese NAFLD patients [25]. The TLL1 SNP was not related to the frequency of histologically evaluated HS in HBV-infected patients (Supplementary Figure S4); however, patients with the major (AA) type had a higher rate of VR than those with other types (Figure 2A). We also found that patients with the AA type showed a statistically significant reduction in HBsAg values, while those with the non-AA types did not (Figure 2B).

**Figure 3:**
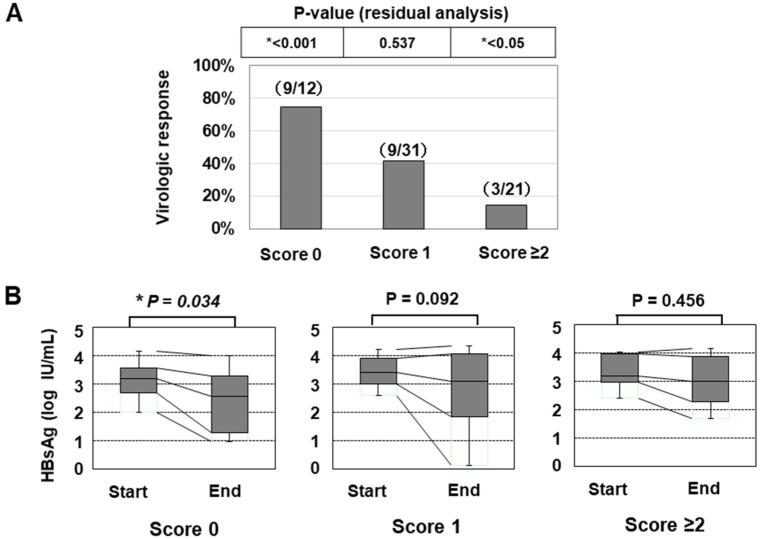
The association of the genetic risk score with the treatment efficacy of PEG-IFN therapy. (**A**) the virologic response rates of patients were shown (*: *p* < 0.05). (**B**) the association of the genetic risk score with the decrease in the HBsAg titer in response to PEG-IFN treatment. The HBsAg values at the initiation and termination of PEG-IFN treatment were compared (*: *p* < 0.05).

In line with a previous paper [25], we also classified the patients according to the number of risk alleles of the two SNPs (G allele of PNPLA3 or T allele of TLL1), and the numbers of patients with 0/1/2/3/4 risk alleles were 12/31/20/1/0, respectively. As only one patient had ≥3 risk alleles, we categorized the patients into the three groups: ‘Genetic risk score 0 (with no risk allele)’, ‘Genetic risk score 1 (with one risk allele)’, and ‘Genetic risk score ≥2 (with two or three risk alleles)’. The classifications of the genetic risk score were significantly associated with the VR ratios (Figure 3A and Table 5). In addition, the HBsAg titer was significantly decreased in the group with ‘score 0′ but not in the other groups (Figure 3B).

Given the favorable response noted among patients with ‘score 0′, we included the genetic score as a clinical characteristic in addition to the factors listed in Table 4 and performed a multivariate analysis. A genetic risk score of 0 and HBeAg positivity were found to be independently associated with the VR (Table 6 and Supplementary Table S2).

Since many non-responders needed to resume NA treatment (including tenofovir), it was not easy to evaluate the long-term changes in the HBsAg titer of all patients after PEG-IFN monotherapy. However, when we assessed the patients who did not receive any antiviral treatment after the off-treatment phase, HBsAg loss (dropping below the lower limit of detection) occurred within 48 weeks in six patients (6/64: 9.3%). None of these patients had histologically confirmed HS. HBsAg loss was observed in three patients in the ‘Genetic risk score 0′ group (3/12: 25.0%) and in three patients in the ‘Genetic risk score 1′ (3/31: 9.7%) group. However, none of the patients with a ‘Genetic risk score ≥2′ achieved HBsAg loss (0/21: 0.0%) (Table 7).

## 3. Discussion

HBV infection is a major health concern worldwide. NAs are excellent drugs for suppressing the replication of HBV, but many patients need to receive long-term treatment. PEG-IFN is a possible therapeutic tool for successfully withdrawing NA treatment in HBV-infected patients, and we participated in two clinical studies on this point in Japan; however, the VR ratios were not high, being around 30% in both [26,27]. We therefore explored new factors associated with the treatment efficacy of PEG-IFN following NA treatment. 

NAFLD is a major lifestyle-associated health concern. However, the effect of HS in antiviral treatment for HBV has varied among studies. For instance, a recent paper suggested that complication with NAFLD/HS was associated with the promising efficacy of anti-HBV treatment in 80 pediatric patients [28], while a recent meta-analysis of studies in adult patients suggested that HS may reduce the efficacy of antiviral therapy for HBV infection [8]. Our data agreed with the recent meta-analysis of studies in adult patients [8] and suggested that NAFLD/HS and its associated gene polymorphisms might be involved in the response to NA/PEG-IFN sequential therapy in HBV-infected Japanese patients.

Unlike HCV infection, the relevance of IL28B SNPs to the treatment efficacy of PEG-IFN in patients with HBV infection remains controversial [29,30], and no genetic factors have been confirmed to influence the effects of PEG-IFN therapy in HBV-infected Asian patients [3]. Our results may provide some new insights into factors predicting the effects of PEG-IFN therapy for HBV infection, although the precise mechanism remains to be clarified. 

Several limitations associated with the present study warrant mention. First, our treatment protocol was designed to administer PEG-IFN therapy to patients who had already been treated with NA (‘sequential therapy’). Although the duration of the preceding NA treatment was not related to the VR (Table 4), and its treatment efficacy was not found to be an independently associated factor (Table 6), our findings cannot be directly compared to those of previous studies regarding PEG-IFN monotherapy without the administration of NA. Second, this study was a retrospective analysis that included 64 patients. When we divided the cases into two groups according to the timing of the initiation of PEG-IFN (N=32, each group), mildly significant differences (*p* < 0.05) were found in some clinical characteristics between the patients in the first-half group and those in the second-half group (Supplementary Table S3). Nevertheless, in both cohorts, the patients with a ‘genetic risk score 0′ had a significantly higher response rate than other groups (Supplementary Table S4), suggesting the clinical relevance of the genetic risk factor to the treatment efficacy. However, the VR rate in the ‘score ≥1′ group was 23.1% (12/52), while the rate in the ‘score 0′ group was 75.0% (9/12) in the current study (Figure 3A). When conducting a new study with reference to these ratios, 15 patients in the ‘score 0′ group should be planned for inclusion (significance level: 0.05, and power: 0.8). Therefore, further validation in another study with a larger cohort is still required in order to draw conclusions regarding the clinical impact of genetic risk factors. Third, the susceptibility to HS may vary among ethnic and geographic backgrounds. Recent studies have also suggested that the clinical impact of TLL1 SNP may differ to some degree between Japanese and Caucasian [24,31]. Thus, the role of PNPLA3 and TLL1 SNPs in HBV-infected patients should be further evaluated using the data from patients outside Japan. A larger-scale study of HBV-infected patients, including NA/PEG-IFN sequential therapy with a defined NA and PEG-IFN treatment schedule, is warranted. Finally, as described above, our research results did not clarify the mechanisms underlying how these gene SNPs affect the efficacy of anti-HBV therapy. The expression of genes located downstream of PNPLA3 and TLL1 should be verified by further experiments to better understand the role of HS and its related genes in HBV-infected patients.

In summary, our results suggested that NAFLD-related SNPs may be associated with not only with the presence of HS but also the efficacy of antiviral therapy in HBV-infected patients.

## 4. Patients and Methods

### 4.1. Patients

Chronic HBV infection was defined as a positive HBsAg status for more than six months. The following exclusion criteria were applied: alcohol intake ≥20 g/day, hepatocellular carcinoma, receiving immunosuppressive therapy, HIV co-infection, and HCV co-infection. The current study was conducted under the approval of the ethics committee of the institutional review board (Nos. 1831, 3321 and Hi-92). Written informed consent regarding the liver biopsy and the research use of the clinical data and genomic analyses was obtained from all of the patients.

### 4.2. Genotyping of Genetic Polymorphisms

Among the genetic polymorphisms associated with HS, we first evaluated the gene polymorphisms PNPLA3 C>G (rs738409) and TM6SF2 C>T (rs58542926), based on a recent review article concerning Japanese NAFLD patients [18]. We also evaluated recently reported NAFLD-related HSD17B13 gene SNPs [19,20,21]. Of the three SNPs with a suggested relationship to NAFLD histology (rs72613567, rs6834314 and rs62305723), rs6834314, and rs62305723 were determined with commercially available kits. For the rs72613567 SNP (T>TA; minor allele: an insertion of an adenine), we used the probe set described in the previous report [21]. In addition, the SNPs of the MBOAT7 C>T (rs641738) and the GCKR SNP C>T (rs1260326) were also determined [21]. We further determined the TLL1 gene SNP (rs17047200), which was shown to be related to the development of HCC in HCV-eradicated patients [23,24] and was recently reported to influence the clinical features in Japanese NAFLD patients in combination with the PNPLA3 C>G (rs738409) SNP [25].

Genomic DNA was isolated from peripheral mononuclear cells and stored until use at −20 °C [22]. The SNPs of PNPLA3 (rs738409), TM6SF27 (rs58542926), HSD17B13 (rs6834314 and rs62305723), MBOAT7 (rs641738), GCKR (rs1260326), and TLL1 (rs17047200) were determined with real-time polymerase chain reaction (PCR) using TaqMan^®^ SNP Assays (Thermo Fisher Scientific Japan, Tokyo: Catalogue No. 4351379; Assay ID rs738409: C__7241_10, rs58542926: C__89463510_10, rs6834314: C__30687619_10, rs62305723: C__89666454_10, rs641738: C___8716820_10, rs1260326: C___2862880_1, and rs17047200: C__33773674_10) according to the manufacturer’s instructions. With respect to the treatment efficacy of the PEG-IFN therapy, we also determined the IL28B gene SNP T>G (rs8099917), which has been reported to be significantly associated with the efficacy of IFN treatment for HCV infection according to the methods previously described [22].

### 4.3. The Liver Biopsy and Laboratory Data

We retrospectively evaluated a total of 224 HBV-positive patients who underwent a percutaneous liver biopsy between August 2010 and June 2017. We histologically estimated the fibrosis stages and the degree of HS, as described previously (histologically confirmed HS was defined as ≥ 5% HS) [4,7]. Recent studies have reported the clinical significance of the coexistence of NASH in HBV-infected patients [32,33]. However, the histological definition of NASH in HBV-infected patients has not been accurately defined, and the Asia-Pacific guidelines exclude HBV-infected patients from the definition of NAFLD [34]. Thus, the present study focused only on the presence of HS. 

The histological findings were externally assessed by expert pathologists without any clinical information (SMC Laboratories, Inc., Tokyo, Japan). In addition to common laboratory variables, given the current clinical importance of quantitative HBsAg [35], we measured the HBsAg titer as well as the HBeAg and HBV-DNA titers [7]. All blood samples were collected on the day of the liver biopsy under fasting conditions.

### 4.4. PEG-IFN Therapy and Its Efficacy

We previously participated in two prospective cohort studies regarding PEG-IFN monotherapy following NA treatment [26,27]. As mentioned in a previous study, the analysis of one study arm was considered to include minimal bias [26]. In the present study, we retrospectively analyzed the pooled patients who had been enrolled in these studies from our institute and whose liver histology had been assessed before PEG-IFN. 

In brief, patients who had been treated with NA for more than one year received PEG-IFN therapy (Chugai Pharmaceutical Co., Ltd., Tokyo, Japan). NA treatment was discontinued within four weeks after the initiation of PEG-IFN treatment, and PEG-IFN (180 μg per body, once a week) was administered for 48 weeks. Patients with a low HBV DNA titer (<4.0 log copies/mL: equivalent to 2000 IU/mL) and HBeAg negativity at 48 weeks after the off-treatment phase were defined as having a sustained VR [3].

### 4.5. Statistical Analyses

Quantitative variables are shown as the median (range). The statistical significance of differences between two groups was determined using the Mann–Whitney U test. In the multivariate analysis, logistic regression models were generated with potential associated factors selected from among those with *p*-values of <0.05. Differences in the frequency between groups were assessed using the chi-squared test or Fisher’s exact test. To compare the frequency among three groups, the group with a significantly higher or lower ratio than the other groups was determined using a residual analysis. To analyze the associations of PNPLA3 genetic variants with the presence of HS and the treatment efficacy of PEG-IFN, the genotypes were entered as a continuous variable (0, 1, and 2 for major homozygotes, heterozygotes and minor homozygotes, respectively), and the linear trend across genotypes was analyzed. To determine the risk score with the combination of the two gene SNPs (PNPLA3 rs738409 and TLL1 rs17047200), we used the group with ‘genetic risk score ≥2′. We therefore entered the classifications of the genetic risk score (score 0, 1 and ≥2) as a categorical variable and determined the odds ratios. In addition, *p*-values of <0.05 were considered to be statistically significant. The JMP 13 software (SAS Institute Inc., Cary, NC, USA) was used for the statistical analysis.

## 5. Conclusions

The PNPLA3 SNP might be associated with the presence of HS, and the combination of the PNPLA3 and TLL1 SNPs might be related to the efficacy of PEG-IFN monotherapy following NA treatment.

## Figures and Tables

**Table 1:** Characteristics of the HBV-Infected Patients who Underwent a Liver Biopsy (*n* = 224).

Gender (Male/Female)	128/96
Age (years)	45 (18–78)
Body Mass Index	22.7 (16.3–42.7)
AST (IU/L)	24.5 (11–242)
ALT (IU/L)	24 (7–513)
GGT (IU/L)	22 (7–349)
ALP (IU/L)	202 (71–655)
Total bilirubin (mg/dL)	0.8 (0.2–2.9)
Albumin (g/dL)	4.0 (2.7–5.1)
Platelets (×10^3^/μL)	186 (43–379)
Prothrombin time (%)	88.7 (57.4–132.3)
Glucose (mg/dL)	89 (71–163)
Triglyceride (mg/dL)	90 (31–333)
Total Cholesterol (mg/dL)	175 (88–311)
HBV Genotype (A/B/C/D/ND)	9/24/174/2/15
HBV-DNA (Log copies/mL)	3.2 (<2.1–≥9.1)
HBeAg (-/+)	158/66
Histological stage of liver fibrosis (F0-1/F2/F3/F4)	126/51/38/9

Quantitative variables are expressed as the median (range). GGT: γ-glutamyl transferase, ALP: alkaline phosphatase, HBV: hepatitis B virus.

**Table 2:** The Relationship of NAFLD-Associated Gene Polymorphisms with Hepatic Steatosis in a Total of 224 HBV-Infected Patients who Underwent a Liver Biopsy.

	Presence of HS		*p*-Value	HS (-)	HS(+)	*p*-Value
				169 Cases (338 Alleles)	55 Cases (110 Alleles)	
PNPLA3 rs738409	CC CG GG	9/68 24/107 22/49	* 3.5 × 10^−4^	Frequency of G allele		* 9.9 × 10^−5^
				137/338	68/110	
TM6SF2 rs58542926	CC CT	43/181 12/43	0.570	Frequency of T allele		0.591
				31/338	12/110	
HSD17B13 rs72613567	TT T/TA TA/TA	24/107 25/102 6/15	0.334	Frequency of TA allele		0.269
				95/338	37/110	
HSD17B13 rs6834314	AA AG GG	24/107 25/102 6/15	0.334	Frequency of G allele		0.269
				95/338	37/110	
HSD17B13 rs62305723	GG GA	49/208 6/16	0.232	Frequency of A allele		0.239
				10/338	6/110	
MBOAT7 rs641738	CC CT TT	31/138 22/79 2/7	0.849	Frequency of T allele		0.391
				67/338	26/110	
GCKR rs1260326	CC CT TT	10/44 20/106 25/74	0.070	Frequency of T allele		0.091
				184/338	70/110	

Please note the following points: There were no patients with the minor type (TT) of the TM6SF2 (rs58542926) SNP in the current cohort. Consistent with the previous report [20], the genotypes of rs72613567 and rs6834314 were completely equal in all patients in the current cohort. There were no patients with the minor type (AA) of the HSD17B13 (rs62305723) SNP in the current cohort. HS: Hepatic steatosis, PNPLA3: patatin-like phospholipase domain-containing protein 3, TM6SF2: transmembrane 6 superfamily member 2, HSD17B13: 17-beta hydroxysteroid dehydrogenase 13, MBOAT7: membrane-bound O-acyltransferase domain containing 7, GCKR: glucokinase regulatory protein. *: *p* < 0.05.

**Table 3:** The Association of the Genetic Variants of PNPLA3 with the Presence of Hepatic Steatosis.

	Odds Ratio (95% CI)	*p*-Value
PNPLA3 rs738409 (single-unit increments)	2.367 (1.517–3.786)	* 0.0001

The genotypes were entered as a continuous variable (0, 1 and 2 for major homozygotes, heterozygotes, and minor homozygotes, respectively), and change in single-unit increments was shown. PNPLA3: patatin-like phospholipase domain-containing protein 3, CI: confidence interval *: *p* < 0.05.

**Table 4:** The Comparison of the Clinical Characteristics of HBV-Infected Patients Based on the Sustained Virologic Response at 48 Weeks After the Off-Treatment Phase.

	Responder (*n* = 21)	Non-Responder (*n* = 43)	*p*-Value
Gender (Male/Female)	11/10	31/12	0.119
Age (years)	40 (26–74)	43 (30–71)	0.238
Treatment period of NA (years)	2.6 (1.0–10.9)	3.30 (1.0–11.3)	0.177
Treatment efficacy of NA (+/-) ^§^	20/1	30/13	* 0.025
HBeAg (-/+)	18/3	20/23	* 3.0 × 10^−3^
HBV Genotype (A/B/C/ND)	1/1/13/0	3/0/32/1	0.987
Significant fibrosis (≥F2) (-/+)	12/9	25/18	0.940
Hepatic steatosis (-/+)	18/3	30/13	0.167
ALT (IU/L) ^¶^	18 (6–43)	19 (8–80)	0.177
Glucose (mg/dL)	83 (79–104)	89 (79–145)	0.530
Triglyceride (mg/dL)	78 (45–128)	87 (37–264)	0.527
Total Cholesterol (mg/dL)	156 (137–253)	163 (118–240)	0.181
IL28B rs8099917 (TT/Non-TT)	15/6	35/8	0.520
PNPLA3 rs738409 (CC/Non-CC)	9/12	8/35	* 0.039
TM6SF2 rs58542926 (CC/Non-CC)	19/2	36/7	0.706
HSD17B13 rs72613567 (TT/Non-TT)	7/14	21/22	0.240
HSD17B13 rs6834314 (AA/Non-AA)	7/14	21/22	0.240
HSD17B13 rs62305723 (GG/Non-GG)	19/2	41/2	0.592
MBOAT7 rs641738 (CC/Non-CC)	13/8	24/19	0.643
GCKR rs1260326 (CC/Non-CC)	3/18	14/29	0.120

Quantitative variables are expressed as the median (range). Responders were defined as those with a low HBV DNA titer (<4.0 log copies/mL: equivalent to 2000 IU/mL) and HBeAg negativity at 48 weeks after the off-treatment phase. ^§^ Treatment efficacy was defined by an HBV-DNA titer lower than the quantitative limit (2.1 log copies/mL) at the initiation of PEG-IFN (after previous NA treatment). ^¶^ ALT values at the initiation of PEG-IFN therapy are shown. ND: Not determined. IL28B: interleukin 28B, PNPLA3: patatin-like phospholipase domain-containing protein 3, TM6SF2: transmembrane 6 superfamily member 2, HSD17B13: 17-beta hydroxysteroid dehydrogenase 13, MBOAT7: membrane-bound O-acyltransferase domain containing 7, GCKR: glucokinase regulatory protein. *: *p* < 0.05.

**Table 5:** The Association of the Genetic Risk Score with the Virologic Response Rates of PEG-IFN Therapy.

Genetic Risk Score	Odds Ratio (95% CI)	*p*-Value
0	1 [Reference]	
1	0.136 (0.229–0.623)	* 0.0102
≥2	0.056 (0.009–0.332)	* 0.0015

The classifications of the genetic risk score (score 0, 1 and ≥2) were entered as categorical variables, and the odds ratios were determined (*: *p* < 0.05). CI: confidence interval.

**Table 6:** Results of a Multivariate Analysis for the Factors Associated with a Sustained Virologic Response at 48 Weeks After the Off-Treatment Phase of PEG-IFN.

Multivariate Analysis	Odds Ratio (95% CI)	*p*-Value
Treatment efficacy of NA (+/-) ^§^	2.594 (0.347–53.18)	0.378
HBeAg (-/+)	5.810 (1.363–34.24)	* 0.016
Genetic risk score (0/≥1) ^¶^	8.666 (1.915–54.26)	* 0.0042

^§^ Treatment efficacy was defined by an HBV-DNA titer lower than the quantitative limit (2.1 log copies/mL) at the initiation of PEG-IFN (after previous NA treatment). ^¶^ The genetic risk score was determined as the total number of risk alleles of the two SNPs (G allele of PNPLA3 or T allele of TLL1). PNPLA3: patatin-like phospholipase domain-containing protein 3, TLL1: tolloid-like 1. *: *p* < 0.05.

**Table 7:** Cases in which the HBsAg Titer Decreased to an Undetectable Level within 48 Weeks After the Off-Treatment Phase.

Case No.	Gender	Age (yr)	HBV Genotype	Fibrosis Stage	Hepatic Steatosis	PNPLA3 SNP	TLL1 SNP	Genetic Score
#1	Male	39	C	F2	(-)	CC	AA	0
#2	Female	42	C	F2	(-)	CG	AA	1
#3	Male	46	A	F2	(-)	CC	AA	0
#4	Male	51	C	F3	(-)	CC	AA	0
#5	Male	28	C	F1	(-)	CG	AA	1
#6	Male	41	ND	F1	(-)	CG	AA	1

ND: Not determined.

## Supplementary Materials

Supplementary materials can be found at https://www.mdpi.com/1422-0067/21/9/3089/s1

## Abbreviations

ALP	alkaline phosphatase
CLD	chronic liver disease
GCKR	glucokinase regulatory protein
GGT	γ-glutamyl transferase
HBsAg	hepatitis B virus surface antigen
HBV	hepatitis B virus
HCC	hepatocellular carcinoma
HCV	hepatitis C virus
HS	hepatic steatosis
HSD17B13	17-beta hydroxysteroid dehydrogenase 13
IL28B	interleukin 28B
MBOAT7	membrane-bound O-acyltransferase domain containing 7
NA	nucleotide/nucleoside analogue
NAFLD	nonalcoholic fatty liver disease
PCR	polymerase chain reaction
PEG-IFN	pegylated-interferon
PNPLA3	patatin-like phospholipase domain-containing protein 3
PT	prothrombin time
SNP	single nucleotide polymorphism
TLL1	tolloid-like 1
TM6SF2	transmembrane 6 superfamily member 2
VR	virologic response
