# Supplementary material for: Possible Relevance of PNPLA3 and TLL1 Gene Polymorphisms to the Efficacy of PEG-IFN Therapy for HBV-Infected Patients
Source: Int J Mol Sci. 2020 Apr 27;21(9):3089. doi: 10.3390/ijms21093089 (PMC7247697; doi:10.3390/ijms21093089)
Supplement: Supplementary file 1 [file ijms-21-03089-s001.pdf]

Supplementary Materials

Supplementary Figures

Supplementary Figure S1.

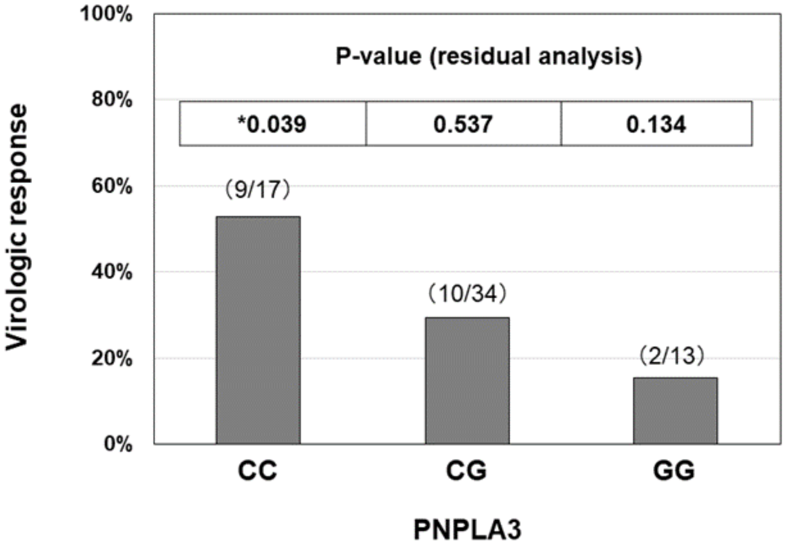

Supplementary Figure S1: Association of PNPLA3 SNPs with the virological response to NA/PEG-IFN therapy. The response rates in patients with the CC, CG and GG types were 52.9% (9/17), 29.4% (10/34) and 15.4% (2/13), respectively. Patients with the CC type had a higher response rate than those with other types (*; $p<0.05$).

Supplementary Figure S2.

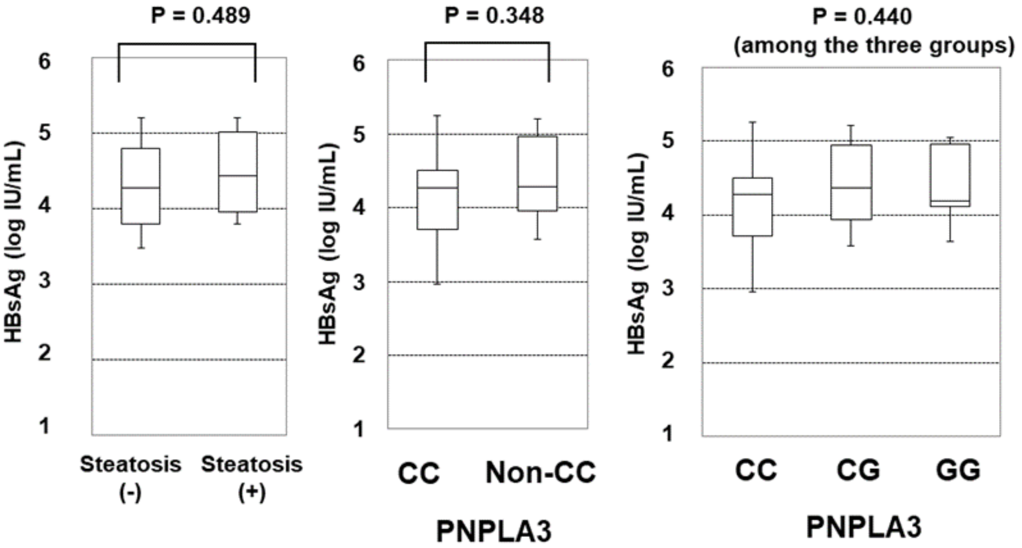

Supplementary Figure S2: Quantitated HBsAg values when switching from NA to PEG-IFN therapy. No significant differences were observed in the quantitated HBsAg values at the initiation of PEG-IFN treatment, regardless of the presence of hepatic steatosis or PNPLA3 SNP.

Supplementary Figure S3.

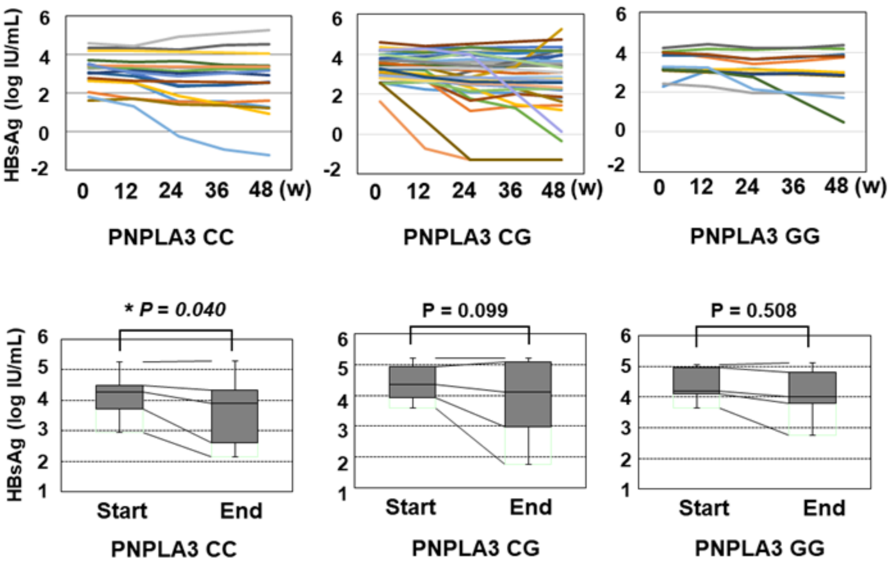

Supplementary Figure S3: Quantitated HBsAg values at the end of PEG-IFN therapy. Regarding the PNPLA3 SNP, there was no significant reduction in the HBsAg titers of patients with non-CC types (see **Figure 2**). Some patients with the CG type responded to PEG-IFN and showed a reduced HBsAg titer, while the reduction of the HBsAg titer was limited in patients with the GG type. *: $p<0.05$

Supplementary Figure S4.

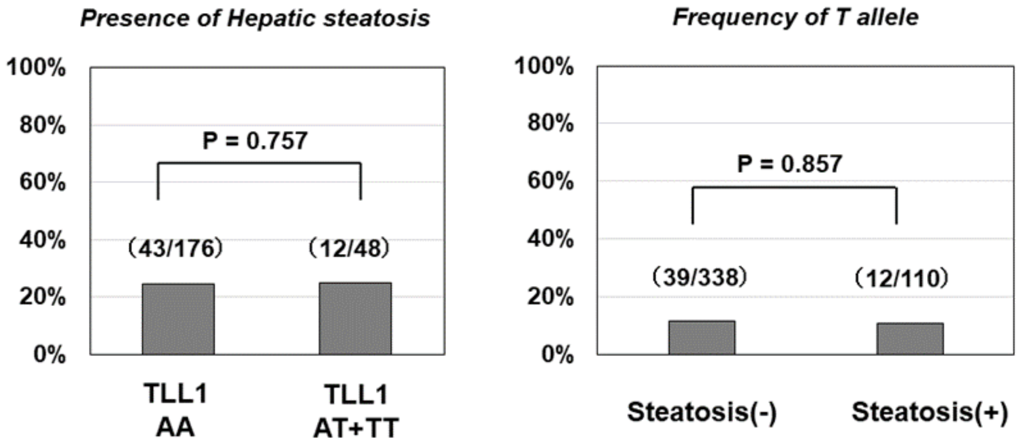

Supplementary Figure S4: The association of TLL1 gene polymorphisms with hepatic steatosis in a total of 224 HBV-infected patients with a liver biopsy. The TLL1 gene SNP was not associated with the frequency of hepatic steatosis in HBV-infected patients (left panel). The frequency of risk alleles did not significantly differ between the patients without and with HS (right panel).

Supplementary Tables

Supplementary Table S1. The association of PNPLA3 SNPs with the virological response to NA/PEG-IFN therapy

	**Odds ratio (95% CI)**	***P* value**
PNPLA3 rs738409 (single-unit increments)	0.393 (0.159-0.886)	*0.0237

The genotypes were entered as a continuous variable (0, 1 and 2 for major homozygotes, heterozygotes, and minor homozygotes, respectively), and change in single-unit increments was shown. CI: confidence interval *: $p < 0.05$.

Supplementary Table S2. The association of the HBeAg-positivity and the genetic risk factor with the virologic response to NA/PEG-IFN therapy

	Virologic response		*p*-value
	Genetic risk score 0	Genetic risk score ≥1	
Total cases	9/ 12 (75.0%)	12/52 (23.1%)	*<0.001
HBeAg (-) cases	8/ 9 (88.9%)	10/ 29 (34.4%)	*<0.01
HBeAg (+) cases	1/ 3 (33.3%)	2/ 23 (8.7%)	0.20

In HBeAg-negative patients, the virologic response (VR) rate was significantly higher in patients with ‘genetic risk factor 0’ than in those with ‘genetic risk factor ≥1’. In patients with ‘genetic risk factor ≥1’, the VR rate was significantly different depending on the HBeAg positivity. These findings suggest that both the HBeAg-positivity and the genetic risk score affected the VR rate.
*: $p < 0.05$

Supplementary Table S3. The clinical characteristics of HBV-infected patients who received PEG-IFN treatment

	First-half group (N=32)	Second-half group (N=32)	*P* value
Gender (Male/ Female)	22/ 10	20/ 12	0.598
Age (years)	43 (26-74)	38.5 (26-71)	0.098
Treatment period of NA (years)	3.4 (1.0-11.3)	1.9 (1.0-10.4)	*0.022
Treatment efficacy of NA (+/-)[§]	24 / 8	26/ 6	0.545
HBeAg (-/+)	20/ 12	18/ 14	0.611
HBV Genotype (A/B/C/ND)	1/ 1/ 28/ 2	3/ 1/ 26/ 2	0.797
Significant fibrosis (≥F2) (-/+)	18/ 14	19/ 13	0.800
Hepatic steatosis (-/+)	24/ 8	24/ 8	1.0
ALT (IU/L)[¶]	21 (8-80)	15 (6-43)	*0.010
Glucose (mg/dL)	88 (79-134)	90.5 (79-145)	0.484
Triglyceride (mg/dL)	80 (40-264)	88.5 (37-206)	0.542
Total Cholesterol (mg/dL)	157 (135-253)	171 (118-250)	0.112
IL28B rs8099917 (TT/ Non-TT)	24/ 8	26/ 6	0.545
PNPLA3 rs738409 (CC/Non-CC)	8/ 24	9/ 23	0.778
TM6SF2 rs58542926 (CC/Non-CC)	27/ 5	28/ 4	1.0
HSD17B13 rs72613567 (TT/Non-TT)	10/ 22	18/ 14	*0.044
HSD17B13 rs6834314 (AA/Non-AA)	10/ 22	18/ 14	*0.044
HSD17B13 rs62305723 (GG/Non-GG)	31/ 1	29/ 3	0.363

MBOAT7 rs641738 (CC/Non-CC)	19/13	18/14	0.800
GCKR rs1260326 (CC/Non-CC)	10/22	7/ 25	0.396

Quantitative variables are expressed as the median (range)

[§]Treatment efficacy was defined by an HBV-DNA titer lower than the quantitative limit (2.1 log copies/mL) at the initiation of PEG-IFN (after previous NA treatment).

[¶]ALT values at the time of the initiation of PEG-IFN therapy are shown.

*: $p < 0.05$

ND: Not determined, IL28B: interleukin 28B, PNPLA3: patatin-like phospholipase domain-containing protein 3, TM6SF2: transmembrane 6 superfamily member 2, HSD17B13: 17-beta hydroxysteroid dehydrogenase 13, MBOAT7: membrane-bound O-acyltransferase domain containing 7, GCKR: glucokinase regulatory protein

Supplementary Table S4. The virological response of HBV-infected patients who received PEG-IFN treatment

	Virologic response		*p*-value
	Genetic risk score 0	Genetic risk score ≥1	
First half group (N=32)	5/ 6 (83.3%)	7/ 26 (26.9%)	*0.019
Second half group (N=32)	4/ 6 (66.7%)	5/ 26 (19.2%)	*0.039

When we divided the cases into two groups according to the timing of the initiation of PEG-IFN (N=32, each group), in both cohorts, the patients with a ‘genetic risk score 0′ had a significantly higher response rate than other groups. *: $p < 0.05$
